# Interacting Timescales in Perspective-Taking

**DOI:** 10.3389/fpsyg.2018.01278

**Published:** 2018-09-10

**Authors:** Rick Dale, Alexia Galati, Camila Alviar, Pablo Contreras Kallens, Adolfo G. Ramirez-Aristizabal, Maryam Tabatabaeian, David W. Vinson

**Affiliations:** ^1^Department of Communication, University of California, Los Angeles, Los Angeles, CA, United States; ^2^Cognitive and Information Sciences, University of California, Merced, Merced, CA, United States; ^3^Department of Psychology, University of Cyprus, Nicosia, Cyprus; ^4^Department of Psychology, Cornell University, Ithaca, NY, United States

**Keywords:** perspective-taking, dynamical systems, interaction, social cognition, joint action, empathy

## Abstract

Through theoretical discussion, literature review, and a computational model, this paper poses a challenge to the notion that perspective-taking involves a fixed architecture in which particular processes have priority. For example, some research suggests that egocentric perspectives can arise more quickly, with other perspectives (such as of task partners) emerging only secondarily. This theoretical dichotomy–between fast egocentric and slow other-centric processes–is challenged here. We propose a general view of perspective-taking as an emergent phenomenon governed by the interplay among cognitive mechanisms that accumulate information at different timescales. We first describe the pervasive relevance of perspective-taking to cognitive science. A dynamical systems model is then introduced that explicitly formulates the timescale interaction proposed. This model illustrates that, rather than having a rigid time course, perspective-taking can be fast or slow depending on factors such as task context. Implications are discussed, with ideas for future empirical research.

## Introduction

Awareness of another's mental states can be key to human survival. The term “survival,” with its evolutionary connotations, belies a modern relevance. But the modern person does routinely face such survival scenarios. For example, imagine riding a bike across several miles of busy traffic to reach work. The coordination of awareness is critical. You glance into the windshields of stopped cars for signs of the drivers' awareness, and to signal your own through mutual gaze, head nods, or facial displays. This awareness and signaling considerably decrease the probability of an accident. This example highlights that perspective-taking continues to be a fundamental skill central to human life. In many everyday situations, people must consider perspectives distinct from their own. These perspectives occur in many common settings, such as giving directions to a visitor, moving furniture with a friend, haggling over a price, and so on. In each circumstance, individuals respond to a large set of psychological states of others, including their knowledge and beliefs, action plans, perceptions or physical viewpoint in space, emotions, and more.

There has been much research on concepts closely related to perspective-taking (Eyal et al., 2018), what cognitive mechanisms underlie it (Brown-Schmidt, 2009a), how sociality and other individual differences are linked to it (Conway et al., 2018), and whether it is a specialized (even inborn) trait (Cohen and German, 2010) unique to humans (Herrmann et al., 2007). Indeed, the concept of perspective-taking is central to many realms of cognitive science. It lies at the heart of many theories of social cognition. As we review below, across different research areas of cognitive science, there is a distinctive debate about the role and nature of perspective-taking.

There are nevertheless identifiable theoretical undercurrents across these different research areas. Perhaps the most prominent undercurrent is the notion of “default perspective” (Nickerson, 1999). The phrase “default process” is sometimes used to describe the first or quickest cognitive state reached (e.g., Lin et al., 2010). There has been debate in several areas of cognitive science between those that favor more or less egocentric processes as holding a kind of “default” status (e.g., Brennan and Hanna, 2009; Shintel and Keysar, 2009). This default status means egocentrism is fast, and most often the first perspective considered. By extension, the default status of egocentrism suggests that considering the perspective of others is slow and effortful. As we will discuss in our review in the section “Pervasiveness of Perspective,” this claim has been made by researchers across different domains of cognitive science. For example, in the study of dialogue, some researchers have explicitly proposed that initial language processing defaults to the speakers' privileged egocentric knowledge, with the conversational partner's perspective being considered later and only if necessary (e.g., Horton and Keysar, 1996; Keysar et al., 1998a,b; Epley et al., 2004).

Having to overcome the egocentric perspective and to ground mutual awareness of another's perspective can indeed be slow and effortful at times. Our introductory scenario is a straightforward example of that: to coordinate that mutual awareness, the cyclist and driver must expend additional effort. Without that effort, they could readily take their own perspective, at potential peril. But despite some evidence for the prominence of the egocentric perspective in some contexts, its default status is still debated. Does the human mind take up its own perspective by default? Or can it default to another's perspective in some situations?

In this paper, we wish to challenge the notion that “fast” and “slow” processes map onto “default” and “secondary” processes associated with egocentric and other-centric information, respectively. Others have also challenged the dichotomy of these clustered features, of fast-default/egocentric processes vs. slow-secondary/other-centric processes. We do not challenge that the egocentric perspective may often be fast to achieve. Instead, we make the argument that, even when considering another person's perspective is “slow,” it need not be secondary and it can have pervasive cognitive impacts. For example, certain tasks can be designed in a way to encourage participants to rapidly take up shared information (Brown-Schmidt, 2012). In some tasks, participants can respond to another's perspective with 100% reliability even when the egocentric response is measurably easier (Duran et al., 2011). Such findings are in line with the view that perspective-taking is a strategic and stabilizing force. It shapes the *dynamics* of our cognitive processing, so that even fast processes can be overcome.

To illustrate our theoretical stance—that perspective-taking is not subject to a single, rigid time course, but is instead adaptive and dynamically fluid—we present a model (section “A Simple Dynamical Systems Model”). This simplified model of perspective is developed in the language of dynamical systems, and integrates these timescales of fast and slow processing. Its details derive from two general observations. The first is that human perspective and perspective-taking depend upon a number of interdependent processes that support each other in the context of some task. We see perspective-taking as a coordinated aspect of human social responding that emerges from the interaction among processes such as perception and attention, working and long-term memory, language processing, and so on. In the language of complex dynamic systems, it is an *emergent process* (Richardson et al., 2014). The second observation is that perspective-taking does not “switch on” in a discrete fashion. Instead, it unfolds dynamically over time in a manner that reflects how this coordination serves social responding. This unfolding over time is an intrinsic aspect of complex dynamical systems (Spivey, 2007), and would be expected from a cognitive process as complex as perspective-taking.

Our simple dynamical systems model illustrates our argument that social perspective-taking may sometimes be *slow*, but this does not necessitate that it be considered *secondary*. We will argue that research on perspective-taking may be furthered by embracing the idea of *interacting timescales*. The principle can be simply stated:

Perspective-taking is driven by interdependent processes that accumulate information at different timescales that should be considered jointly. While shorter timescales can reflect the processes recruited to achieve the first perspective, longer timescales can reveal processes (e.g., those implicated in deliberate social organization) that are recruited to achieve the most stable, target perspective.

Our argument is related to dynamical systems and connectionist approaches to cognition, which see stable performances as an interplay among multiple constraints and processes (Rumelhart and McClelland, 1986; Thelen and Smith, 1994; Port and Van Gelder, 1995; Thelen and Bates, 2003; Van Orden et al., 2003; Spivey, 2007). In the same way, an unfolding task requiring a *perspective* involves establishing a strategy. This strategy tends to stabilize. It is held steady while humans carry out that task. As a task is established, sustained, and completed, multiple cognitive processes—some fast, some slow—interact to bring that strategy into being.

Further below, we describe a model that instantiates in an explicit manner the theoretical commitments we endorse. Before delving into the details of this approach, we introduce a definition of “perspective-taking” on which the theoretical discussion will be based. Following this, we review several areas of social and cognitive science in which perspective-taking is a central process. We then present an implemented dynamic model of perspective-taking, and link it to our prior discussion. To conclude, we argue that the approach makes a number of broad predictions that may guide future work.

## Definitions and mechanisms of perspective-taking

Many areas of cognitive science invoke some notion of perspective and perspective-taking, if sometimes implicitly. In order to clarify what we mean by different cognitive mechanisms, and to illustrate the distinction between “fast” and “slow” on the one hand, and “default” and “secondary” on the other, we offer the following taxonomy of cognitive mechanisms. Cognitive processes underlying perspective can be projected onto a spectrum from simpler to more complex, faster to slower, primary (or “default”) to more secondary. These are illustrated in Table 1.

**Table 1:** A spectrum of cognitive processes from simpler to more complex, with illustrations of each under various domains in which perspective-taking is central.

**Domain**	**Potentiation**	**Emulation**	**Simulation**
	*Simpler faster*	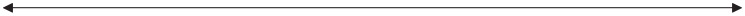	*Complex slower*
Joint action	Co-activating candidate objects and actions for a task from copresence and observation	Predicting probable partner movements from observed perceptuomotor dynamics and affordances	Predicting partner processes from goal-orientation of partner, strategic analysis of local cues from partner and relevant task features
Empathy	Co-activating emotional states from associations of behavioral or environmental accompaniments	Overlapping emotional expression through similar neural circuits and physiological processes (e.g., mimicry of facial expressions)	Recognizing emotional states through appraisal of situational factors and cues
Dialogue	Co-activating linguistic levels of analysis via mere exposure (priming)	Anticipating linguistic levels of analysis through common processes between interlocutors	Inferring and tracking partner processes from strategic combination of linguistic levels, recall of dialogue history, situational cues, etc.
Theory of mind	Co-activating partner's mental states independently of egocentric goals	Identifying partner's mental states through inhibition of egocentric goals and use of executive function	Inferring and tracking partner's mental states from goal orientation of partner and relevant task features

At one end of this spectrum, *potentiation* refers to the rapid inducement of certain cognitive states that can happen by mere exposure or priming in context. Examples are given in the table. In the study of dialogue and language, for example, two people can establish the effects of each other's linguistic conditions merely by exposing each other to a co-activation of certain words, phrases, semantics, etc. (Pickering and Garrod, 2004).

At the other end of this spectrum are slower and more cognitively complex processes, which we will term *simulation* (cf. Lillard, 1998). This term connotes the relatively more effortful cognitive reconstruction of another person's states or processes. It reflects a more strategic means of establishing these effects. In language and dialogue, for example, a person may deliberately recall personal details, dialogue history, topic transitions, etc. that may demand more effortful memory cueing and retrieval. This information may then be deployed even as an element of a conscious strategy (Horton and Gerrig, 2016).

But what counts as taking a perspective in the context of this spectrum? Perspective-taking could be defined simply as adopting *any* conditions of another, typically a conspecific, in order to coordinate actions. This definition may seem too general, as it characterizes even the simplest responsiveness among organisms as “perspective-taking.” Here, our focus is on the human cognitive mechanisms underlying perspective, which we review and model. A definition more specific to human cognition would make reference to the conditions that underlie a perspective or perspective-taking event in our species. Let's hazard a specific defining statement of perspective-taking:

Perspective-taking can be defined as the integration of perceived conditions influencing *another person's* behavior into the set of conditions influencing *one's own* behavior. And a perspective is a subset of those conditions or *possible* conditions that can be coordinated, across a variety of core mental or bodily processes (perceptual, emotional, epistemological, etc.).

This definition embraces many domains in which a perspective may be taken, from spatial cognition to emotional contents. It also asserts that humans sometimes integrate *possible* perspectives of others. This implies we are sometimes incorrect about perspectives, but also that we can anticipate perspectives before others even have them (cf. Ramnani and Miall, 2004).

There are different means by which the human cognitive system integrates perspective. Perspective-taking can sometimes be rather effortful, and so the goal-oriented description is apt (Lin et al., 2010). It may sometimes be more implicit. There is considerable debate about implicit social processes, but there is some evidence that very subtle social variables of one person, such as their mere presence, can alter cognitive processing of another person without their awareness (Golland et al., 2015). For example, as we review below, one person can engage in emotional appraisal to perspective-take the state of another—but this need not induce the very emotions themselves. In other situations, such perspective-taking may indeed induce common emotions.

These subtle distinctions suggest that different combinations of cognitive processes can be involved in specific perspective-taking events. What is key to our theoretical position and dynamical model is that these processes can involve interacting timescales at which information about another's perspective accrues. This interplay permits stable perspective strategies to emerge in varied situations. Perspective-taking could thus be framed as a highly robust feature of our species, deployed dynamically in social tasks. By “robust,” we mean a kind of behavioral repertoire that has multiple mechanisms (cognitive, perceptual, and motor mechanisms) to sustain that repertoire despite variable circumstances and perturbations (Kitano, 2004). These diverse mechanisms appear to be tuned to “the other,” to conspecifics in the social context (Lieberman, 2013; Schilbach et al., 2013).

Below we offer a brief review of each the domains shown in Table 1. The review reveals that perspective-taking is central in each. In each area, we further illustrate how this general spectrum of cognitive processes influences research and debate in these literatures.

## Pervasiveness of perspective

As we have argued above, perspective-taking is central to many areas of cognitive science. Despite the uniqueness of each area, there are theoretical undercurrents common to them. One is a tension between a faster self-oriented perspective, and perspectives that *may* require more time and mentalizing. We consider four domains if research illustrating this: joint action, empathy, human linguistic interaction, and theory of mind. These areas illustrate the broad relevance of human perspective-taking. They also reinforce the need to integrate timescales in our understanding of the dynamics of perspective-taking, especially the intuitions presented in Table 1.

### Joint action

Imagine you have to move a very heavy table from one end of the room to the other. You recruit a friend to help. Your bodies rapidly orient themselves relative to the table. You time your movements appropriately so as not to drop the table. You may also talk openly to each other to shape your behaviors. Such joint actions involve coordination, planning, communication and actions among multiple agents. Their complexity at various timescales is a growing field of study^1^. Perspective-taking is critical in this domain. Moving a table with another person requires responsiveness to physical variables, such as body position and speed, but also psychological ones, such as expectations of the task itself.

Some influential theories of joint action depend on complex cognitive conditions of intention and planning (for discussion see Tollefsen and Dale, 2012). However, recently, joint action has been theorized to involve not only planned but also emergent coordination (Knoblich et al., 2011; van der Wel et al., 2015; Vesper et al., 2017). Emergent joint action does not rely on shared plans, but on entrainment, perception-action couplings, or common affordances for co-actors (Marsh et al., 2009; van der Wel et al., 2015). Humans can respond rapidly using “shallow” coordination processes, with each partner serving as perceptuomotor *affordances* for the other. Many of these shallow processes, along the potentiation end of the spectrum, may not require awareness. But this cannot be the whole story. More strategic mechanisms can quickly constrain a joint action. For example, the sudden appearance of a mere syllable by a friend—“ouch”—can elicit a cascade of cognitive effects in others. In addition to strategic adjustments to such local cues, top-down factors, such as shared knowledge, can constrain the emergent coordination of interacting partners (e.g., their eye-movements: Richardson et al., 2007).

### Empathy

In social psychology, extensive studies of shared emotional states and their social implications have been conducted over the past few decades (Hatfield et al., 1993), including in social cognitive neuroscience (Singer and Lamm, 2009). Empathy involves sharing other people's emotional experiences, while also being able to represent the other as the source of the emotion (Decety and Jackson, 2006; De Vignemont and Singer, 2006). We could consider empathy as a kind of “emotional perspective-taking” (Davis, 1983; Gehlbach, 2004; Lamm et al., 2008).

Both automatic and more deliberate processes appear to be at play, as shown along the spectrum shown of Table 1. In fact, these processes can be seen as distinguishing between affective and cognitive components of empathy. The affective component is typically thought to rely on automatic processes triggered by mere social-emotional stimuli. For example, non-conscious mimicry of others' facial expressions, postures and so on gives rise to phenomena such as emotional contagion (Hatfield et al., 1993). The cognitive component, recruited in the appraisal of people and situations, is thought to rely on more controlled processes that generate or modify affective responses (e.g., Preston and Hofelich, 2012; Cuff et al., 2016; Vinson et al., 2017).

Some research corroborates this distinction, with findings that automatic and more controlled aspects of empathy operate at different timescales, and in context-sensitive ways (De Vignemont and Singer, 2006; Zaki, 2014). Additionally, some ERP tasks suggest that affect sharing is detected quickly, and cognitive appraisal of a painful situation is detected a few milliseconds later (Fan and Han, 2008).

Like joint action, empathy has presented a tension between automatic vs. strategic processes, and deep vs. shallow social information (e.g., how much of others' affective experience is neutrally represented: Singer et al., 2004). These tensions can cloud the fact that empathy is shown to be multiply constrained by different contextual and cognitive variables (cf. Davis, 1983; Gehlbach, 2004). Consistent with our view of perspective-taking, empathy is unlikely to involve a rigid time course; instead, relevant social and affective information can be integrated through interacting timescales.

### Linguistic interaction

Dialogue is another uncontested site of human perspective-taking, as interlocutors frequently consider one another's informational needs. One central question is how interlocutors infer and keep track of each other's perspective. In terms of inferring a conversational partner's perspective, some researchers claim that it is derived from basic memory representations about prior shared experiences (Horton and Gerrig, 2005), or from information in the immediate shared physical environment and ongoing interaction (Clark, 1996). In terms of keeping track of that perspective, one proposal is that it can be represented in terms of simple (often binary) distinctions (Brennan and Hanna, 2009; Brennan et al., 2010). For instance, speakers can keep track of whether the partner has heard a story before or not (Galati and Brennan, 2010), whether a particular category of items has been discussed with the partner or not (Horton and Gerrig, 2005), and so on. When the communicative situation supports such a simple distinction about perspective overlap, speakers can keep track and cue that distinction, adjusting their language use appropriately.

There's nevertheless a lively debate concerning how quickly speakers can take their partner's perspective into account during language processing. As we have noted earlier, one view in this debate is that early processing gives priority to egocentric information, with the partner's perspective being considered late. Keysar and his colleagues describe this in terms of the two-stage model of *anchoring and adjustment*, whereby initial processing defaults to egocentric information, without regard to the partner's perspective or informational needs; the products of this initial processing are monitored and partner-specific adjustments are made only when necessary, in the form of repairs (Horton and Keysar, 1996; Keysar et al., 1998a,b). In a related proposal, speakers are thought to align different levels of their linguistic representations through the low-level mechanism of priming (i.e., potentiation) (Pickering and Garrod, 2004). In this view, potentiation scaffolds adaptation in dialogue without speakers having to track anything specific about the partner's perspective. The competing view in this debate is that information pertinent to the partner's perspective can shape early processing (Hanna et al., 2003; Metzing and Brennan, 2003; Brown-Schmidt and Tanenhaus, 2008) when available and easily tracked (Galati and Brennan, 2010).

Much like empathy and joint action, both rapid processes and more strategic ones are likely involved in dialogue, operating at different timescales, depending on task conditions and availability of memories (Horton and Gerrig, 2005) or certain cognitive processes (e.g., executive function: Brown-Schmidt, 2009a).

### Theory of mind

Humans make complex social judgments by inferring partner knowledge, and predicting potential behaviors and beliefs. Some researchers propose that such skills invoke a *theory of mind* (ToM), the ability to attribute and reason from mental states of others, while distinguishing them from one's own (Flavell, 1999)^2^.

One account about the representation of others' psychological states is Butterfill and Apperly's *minimal theory of mind* (Butterfill and Apperly, 2013). This account is in line with proposals from the previous domains, advocating for minimal representational requirements for joint action (Vesper et al., 2010) and simple models of the partner in dialogue (Brennan et al., 2010). According to this account, people represent simpler, relational mental states (e.g., goals to which actions are directed), which enable them to keep track of others' propositional attitudes, such as beliefs, without representing them fully as such. Such minimal representations are thought to eliminate the conceptual and cognitive demands associated with representing fully others' mental states. However, there is some debate: some have argued that social awareness of a rich sort is based on innate or specialized capacities that permit more complicated knowledge (see discussion in Cohen and German, 2010; Mazzone, 2015).

As with the previous domains, tracking the beliefs of others seems to be triggered by both simpler, rapid processes, or more involved mentalizing. For instance, tracking others' beliefs or perceptions can occur independently of their relevance to the task (Kovács et al., 2010; Samson et al., 2010). On the other hand, tracking others' beliefs is influenced by demands on executive functioning, which can be taken as evidence of more controlled processes. Inhibitory and executive control functions are important mechanisms for ToM: for successful perspective attributions, one must inhibit their egocentric perspective and prevent that perspective from interfering with apprehending the perspectives of others (Samson et al., 2005). Thus, findings that people incur a cognitive processing cost when they consider others' perceptions or beliefs (Apperly et al., 2008) or that such consideration taxes executive function (Apperly et al., 2004; Samson et al., 2005) can be thought to reflect more strategic consideration of others' mental states.

As with the other phenomena reviewed in this section, ToM is not merely a unitary or static process whereby people hold a body of knowledge about others' beliefs, but is rather a dynamic one, greatly integrated with other processes, including decision-making and executive control. This position is consistent with Christensen and Michael (2016), who have recently argued that mindreading of this kind is better understood as a multi-system architecture rather than a fixed two-system approach. Rather than a minimal two-system account, they offer one that integrates causal reasoning, knowledge, and so on, and attempt to capture a wider array of infant and adult findings.

## A simple dynamical systems model

These four domains are not often regarded as integrated, even though they intuitively lend themselves to an integrative stance^3^. This intuitiveness derives from a shared feature: each domain centrally involves what may be termed “other-centrism.” Whether an experimental task is of joint action, empathy, linguistic interaction, or theory of mind, participants must track something about another person in some manner. The thread of perspective-taking weaves these domains naturally. There is even a related theoretical tension common across these domains concerning the complexity of the other-centric “representations” tracked in perspective-taking. Across domains, there's a debate about the extent to which people explicitly track detailed perspectival information (e.g., belief structures) about a task partner (e.g., Shintel and Keysar, 2009; Butterfill and Apperly, 2013).

Moreover, as our review makes evident, in all these domains, perspective-taking does not unfold over a single rigid time course and is not mechanistically unitary. Perspective-taking and mutual responsiveness between task partners can be supported by quick and associative processes, but also by more strategic, inferential processes. Perspective-taking can be seen as being supported by many *different* core mechanisms, including automatic and controlled processes (Lieberman, 2007).

An important next step in theory and model development is, therefore, to articulate the manner in which faster and slower mechanisms interact and how perspective responses emerge dynamically from mechanisms shaped by task constraints. It cannot simply be the case that one mechanism dominates over others in each circumstance. Perspective responses are dynamic strategies emerging from *multiple and mutually interacting* processes that influence the human responder as a task unfolds.

We take a simple approach here, by using a dynamic systems model, adapted from Duran and Dale (2014). Some models are meant to capture basic theoretical distinctions more explicitly, and leave out a variety of concrete details (McClelland, 2009). Our model here is of this kind. It offers explicit formalization of our claims. We aim to map out the dynamic relationship among processes on the spectrum illustrated in Table 1. We do not mean to imply that processes along this spectrum are independent or encapsulated, as in a module. Instead, they interact. The model here captures an interplay between these processes in a simplified “perspective” task. Though the model is extremely simple, it serves to illustrate how tasks and processes interact to move perspective-taking around.

### Form of the model

First, we briefly describe the model developed in Duran and Dale (2014). They imagine a simple task in which a participant decides between two alternative perspectives. Perspective is represented by a numeric variable *x*. Values of *x* are assigned a particular interpretation by the modelers. In our case, *x* varies from negative to positive numbers and we assign these positions ego- vs. other-centric orientation, respectively. We also assume perspective is a continuously evolving state. Gradations of state variables are common in cognitive models such as neural networks or dynamic systems (Spivey, 2007). A competition between two opposing states can also be approximated as a gradient (Onnis and Spivey, 2012). It is the *dynamics* of the system that matter, though, and we assume that the system has two stable states of value *x*. Duran and Dale (2014) use the following model from Tuller et al. (1994) to capture this:

(1) $$\begin{array}{r} V=kx-\frac{x^{2}}{2}+\frac{x^{4}}{4} \end{array}$$

**Figure 1:**
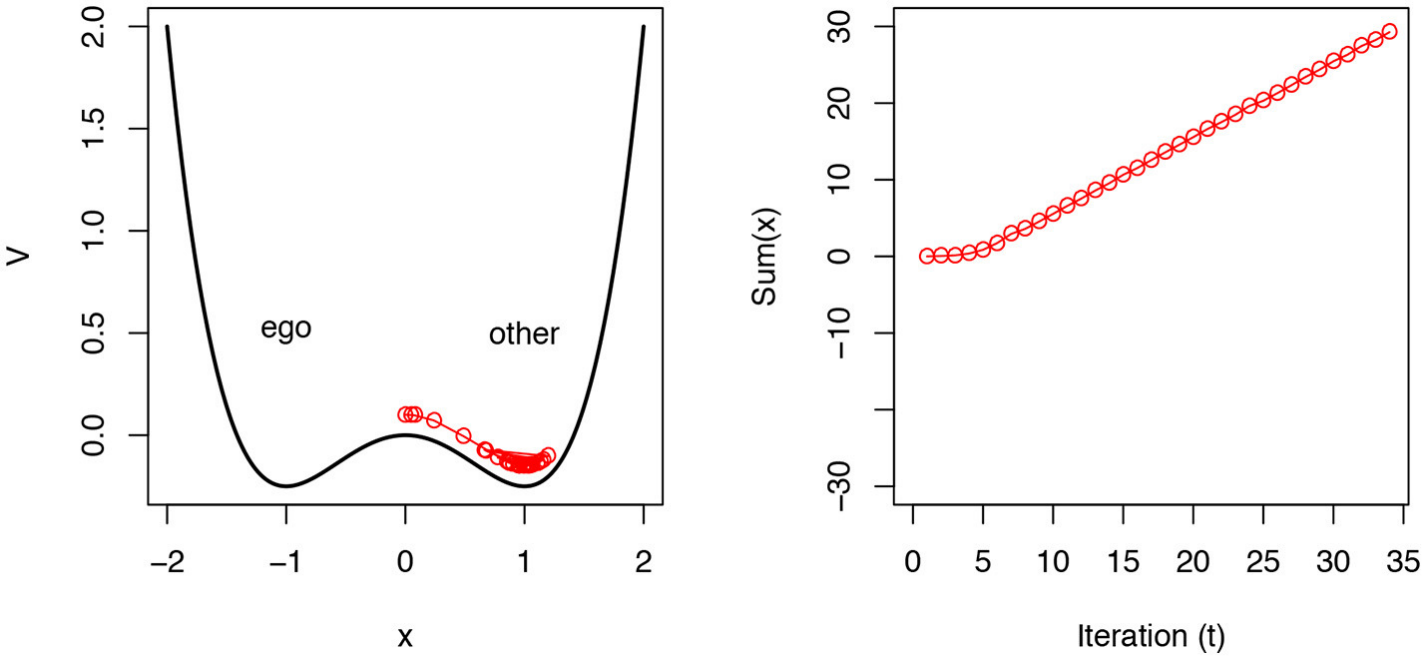
The potential *V* defined in Equation (1) is illustrated on the left. Here, *k* is 0. The red line is an illustration of how a simulated “trial” is run in the model. The model is initialized near the saddle point (at 0), and it acts as a kind of biased drift/diffusion process as it settles into an assigned interpretive role, defined by Equation (2). On the right, we show that this “decision” is achieved at a threshold sum (∑*x*). In Duran and Dale (2014), several features of perspective-taking timing were modeled with this basic mechanism.

This equation is called the *potential* of the system. We can assume that a state variable *x* will settle into one of the local minima of this equation, illustrated in Figure 1, left. Duran and Dale (2014) model perspective-taking under this formulation by assigning one side of this model to “ego” and the other to “other-centric” perspectives, and explore the consequences of various task parameters. An important control parameter is *k*, which determines the “tilt” of the system and can bias a system toward one perspective or another. These and other subtle parameters allow the researcher to set these interpretations of the model's dynamics and map them onto explicit predictions about the timing of perspective-taking.

Duran and Dale (2014) fit several features of experimental perspective-taking data. They focused especially on dynamic features of data, derived from listeners' mouse trajectories in the perspective-taking task. They manipulated this simple model in the following way. If we take a single trial of an experiment as a process of observing perspective (*x*) settle into one minimum or another, we can define the following update equation and observe how the system dynamically fluctuates and settles into an attractor. Notice that the update is based on the first-order derivative of *V* above:

(2) $$\begin{array}{r} x_{t+1}=x_{t}+\left(-k+x_{t}-x_{t}^{3}\right)+N\left(0,\sigma \right) \end{array}$$

*N*(0, σ) is a source of Gaussian noise of mean 0 and standard deviation σ. This makes this model similar to a dynamic diffusion process, where the *x* state variable moves toward one threshold or another (Ratcliff et al., 1999). In Duran and Dale (2014), they maintain an accumulator (∑*x*) and when this accumulator reaches a threshold (e.g., 30) the system is taken to have fully settled on one side or another. This is illustrated in Figure 1, right. Interestingly, with this setup, Duran and Dale (2014) were able fit three timescales of responses: (i) the time to a perspective-taking decision within a trial, (ii) the change in response timing across trials, and (iii) the overall response strategy of subjects in a single set of parameters.

### Expanding the model: two processes

The proposal in this paper is that perspective-taking is not driven by a single underlying process, but by a synergy of processes that are operating at different timescales. These processes interact and produce the kinds of perspective-taking choices we see in our experiments and in everyday life. We can simulate that simply here to bring some explicitness to our proposal. This model can also serve as a foundation for follow-up work that combines it with new experimental data in a similar manner to Duran and Dale (2014)^4^.

For simplicity, let's take perspective-taking to be driven by two information accumulation processes, *x*_*P*_ and *x*_*S*_, meant to capture the distinction between leftmost and rightmost columns of Table 1. As described above, we can take one of these processes (*x*_*P*_) and take it to reflect the automatic, rapid establishment of a decisions (“P” for “potentiation”). We can take the other (*x*_*S*_) as a slower more deliberative process (“S” for “simulation”). The slower more deliberative process may accumulate more slowly, but it can have a deeper attractor well—an established strategy can thus “pull” the simpler processes toward that decision (even, perhaps, when the faster information violates it).

To update these two systems, we will combine the basic information in the prior model formulation of Duran and Dale (2014). We will also integrate an interactive parameter—if information of both processes supports each other, this may facilitate establishment of a response. We modify this model slightly in the following way:

(3) $$\begin{array}{r} x_{P,t+1}=x_{P,t}-u_{P}\overset{\cdot }{V}\left(k_{P,t}\right)+\alpha \left(x_{S,t}-x_{P,t}\right)+N\left(0,\sigma \right)\\ x_{S,t+1}=x_{S,t}-u_{S}\overset{\cdot }{V}\left(k_{S,t}\right)+\beta \left(x_{P,t}-x_{S,t}\right)+N\left(0,\sigma \right) \end{array}$$

Here, $\overset{\cdot }{V}$ represents that first-order derivative shown in the prior section, relative to *x*. Note also that the *difference* between the processes is part of the update equation. If the current state of the high-level process (*x*_*S, t*_) is *higher* than that of the lower process, it will “pull” the system toward it, according to the control parameter α. This relationship is reciprocal, but we can vary the relative strength of this relationship using parameters α and β. In addition, importantly, we can vary the rate at which these systems “descend” upon their perspective choice, using the parameters *u*_*P*_ and *u*_*S*_. Strategic, higher-level systems are slower, and so we can set this parameter smaller than the one for the faster process. We summarize the variables in Table 2.

**Table 2:** The parameters used for Figures 2–5 under Equation (3).

**Figure**	***x*_*p*_**		***x*_*s*_**
	**Simpler Faster**	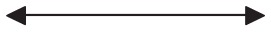	**Complex Slower**
Figure 2	*k*_*p*_ = 0		*k*_*s*_ = 0
(σ = 0.01 for all)	*u*_*p*_ = 0.2		*u*_*s*_ = 0.2
	β = 0		α = 0
Figure 3	***k*_*p*_ = 0.2**		*k*_*s*_ = 0
	*u*_*p*_ = 0.2		***u*_*s*_ = 0.1**
	β = 0		α = 0
Figure 4	*k*_*p*_ = 0.2		***k*_*s*_ = −0.2**
	*u*_*p*_ = 0.2		*u*_*s*_ = 0.1
	β = 0		**α = 0.2**
Figure 5	*k*_*p*_ = 0.2		***k*_*s*_ = −0.025**
	*u*_*p*_ = 0.2		*u*_*s*_ = 0.1
	**β = 0.01**		α = 0.2

*In bold along some rows we highlight key changes from figure to figure. These parameters warp the response landscape, and change the perspective-taking dynamics. For code, see http://github.com/racdale/simple-perspective-model*.

**Figure 2:**
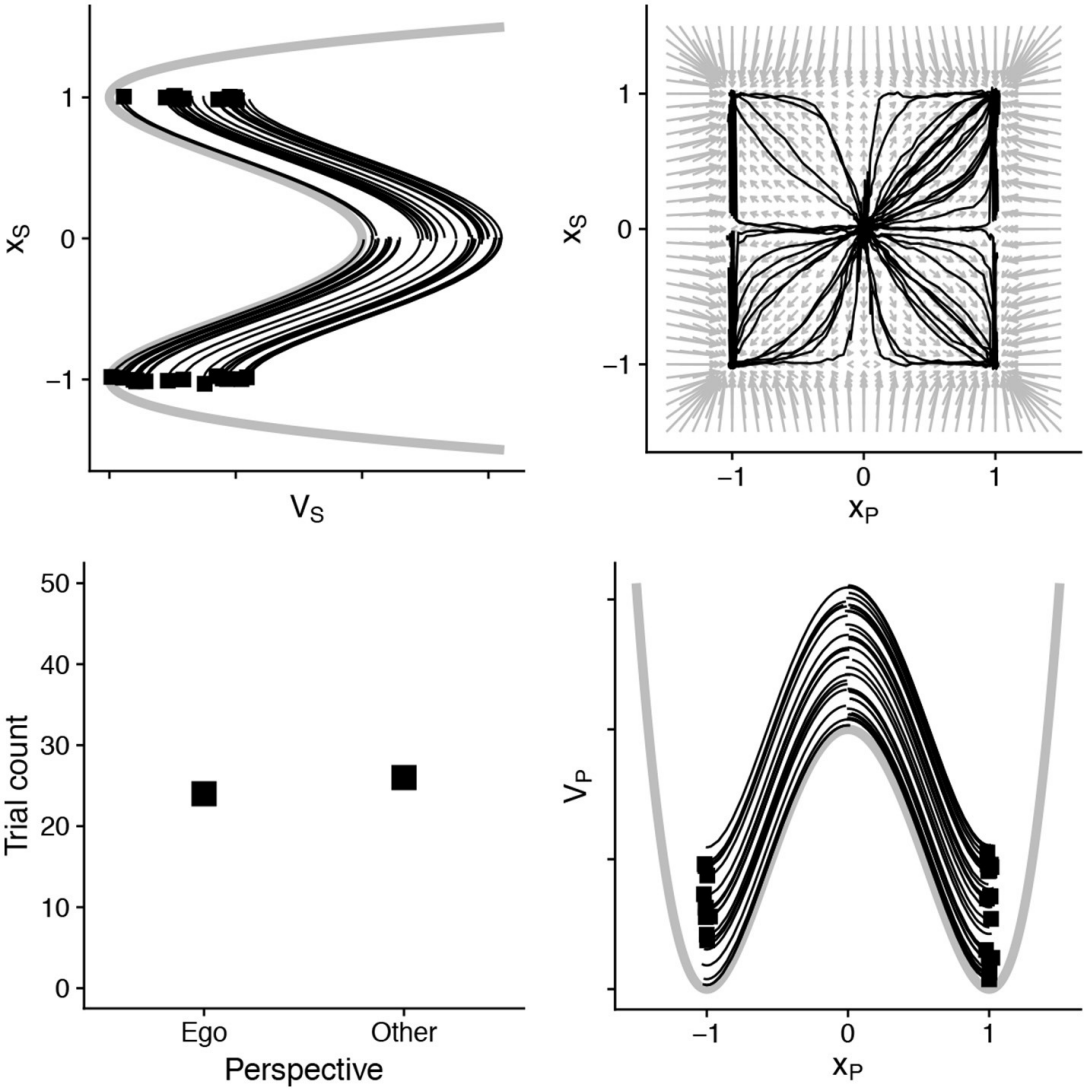
An illustration of the basic two-dimensional dynamical model. On the top left and bottom right are the potential wells of the two variables. These variables evolve as in Equation (3), and together they define a vector field shown in the top right. In the black lines we have simulated 50 “decisions” which show an equibiased perspective response. This is based on the parameters: *u*_*P*_ and *u*_*S*_ = 0.2, *k* for both and α and β set to 0. The noise parameter σ = 0.1. We use a threshold of 30 (see Figure 1), and the squares reflect which state variable (*x*_*S*_ or *x*_*P*_) reached the threshold first. These are summarized in the Table 2. All source code can be downloaded from http://github.com/racdale/simple-perspective-model.

In Figure 2 we show the basic two-dimensional model at work. Because this figure has the same structure throughout our demonstrations, we explain it in detail here. The specification in Equation (3) defines how two processes evolve over time. We can therefore plot them on a two-dimensional plot, and assess the probability that they will move or “flow” in particular directions. This is shown in the top right portion of Figure 2, with the vector field diagram. The arrows represent the tendency for this two-dimensional system to change at particular points over that field. In the top left and bottom right panels are the corresponding fields for each variable, placed over their corresponding axes. Superimposed on top of these three plots we show 50 simulated “decisions”—letting these variables evolve until one has reached a threshold. The distribution of responses is shown in the bottom left part of the plot. In this first simulation, we specify the two processes *x*_*P*_ and *x*_*S*_ as essentially equal in timescale and strength. When doing so, of course, we see an even split between “ego” and “other” perspectives that emerge in the plot.

The parameter *k*_*S, t*_ represents the current “tilt” of the dynamic system for that process. This is illustrated in the example simulations below. In the original application of Duran and Dale (2014), they assumed that the tilt would slowly shift over a series of trials in a simulated experiment; this reflects the incremental strengthening of a strategy. We choose to ignore this aspect of the model here, so as not to add further complexity or free parameters. It is kept in the formulation above to illustrate that other features of this model—across trials rather than just within trials—may also be interesting to explore.

These state variables, though they interact positively, can also compete. We take the perspective “decision” of this model to be when the state variables have accumulated to some level, akin to Duran and Dale (2014). So, at time (*t*) increments, we take an incremental sum of both state variables. When the first reaches some threshold (here, 30) we consider the model to have achieved a stable perspective for that trial. Again, this is akin to threshold drift-diffusion models.

With this basic setup we can simulate a series of basic ideas from the perspective-taking literature. The purpose of the example simulations is to show an idealized dynamic process that establishes perspective through different processes that accumulate information at distinct timescales.

### Illustrating the model

**Figure 3:**
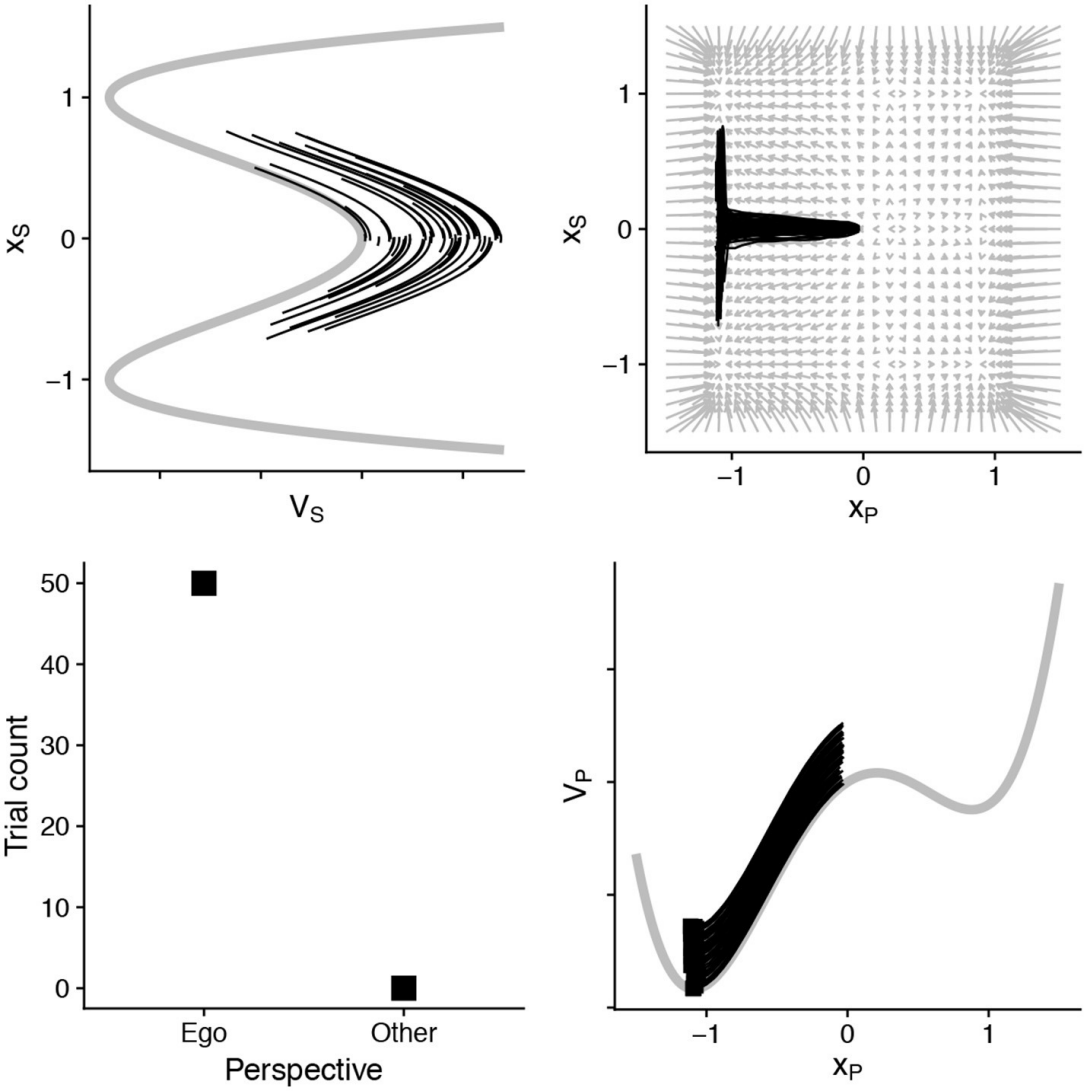
By assuming that the slow (*x*_*S*_) process is half the pace of information accumulation as the faster (*x*_*P*_), and also that the fastest is robustly egocentrically biased—egocentric perspective decisions dominate, and information accumulation in the slower process (shown in top left) does not reach the threshold in any of these 50 simulated “decisions.” See Table 2 for full parameters.

Though surely simple, the model illustrates what we mean by the interaction among timescales explicitly. Consider a basic demonstration: A fast shallow process (*x*_*P*_) will dominate over slower, other-oriented ones (*x*_*S*_). In Figure 3, we show that when we change the values for *u*_*P*_ and *u*_*S*_ the model's behavior changes considerably. We set *u*_*P*_ to be twice the value of *u*_*S*_. This would indicate that the low-level process (*x*_*P*_) accumulates information at twice the speed. When this happens, the model consistently responds egocentrically—the information accumulates too quickly for the slow process to reach its own threshold. The model also demonstrates that when cognitive or task parameters change, the *landscape* for the dynamics changes. The top right panel in Figure 3 shows that the vector field now favors a particular outcome, and even under stochasticity the model will descend toward an egocentric response.

**Figure 4:**
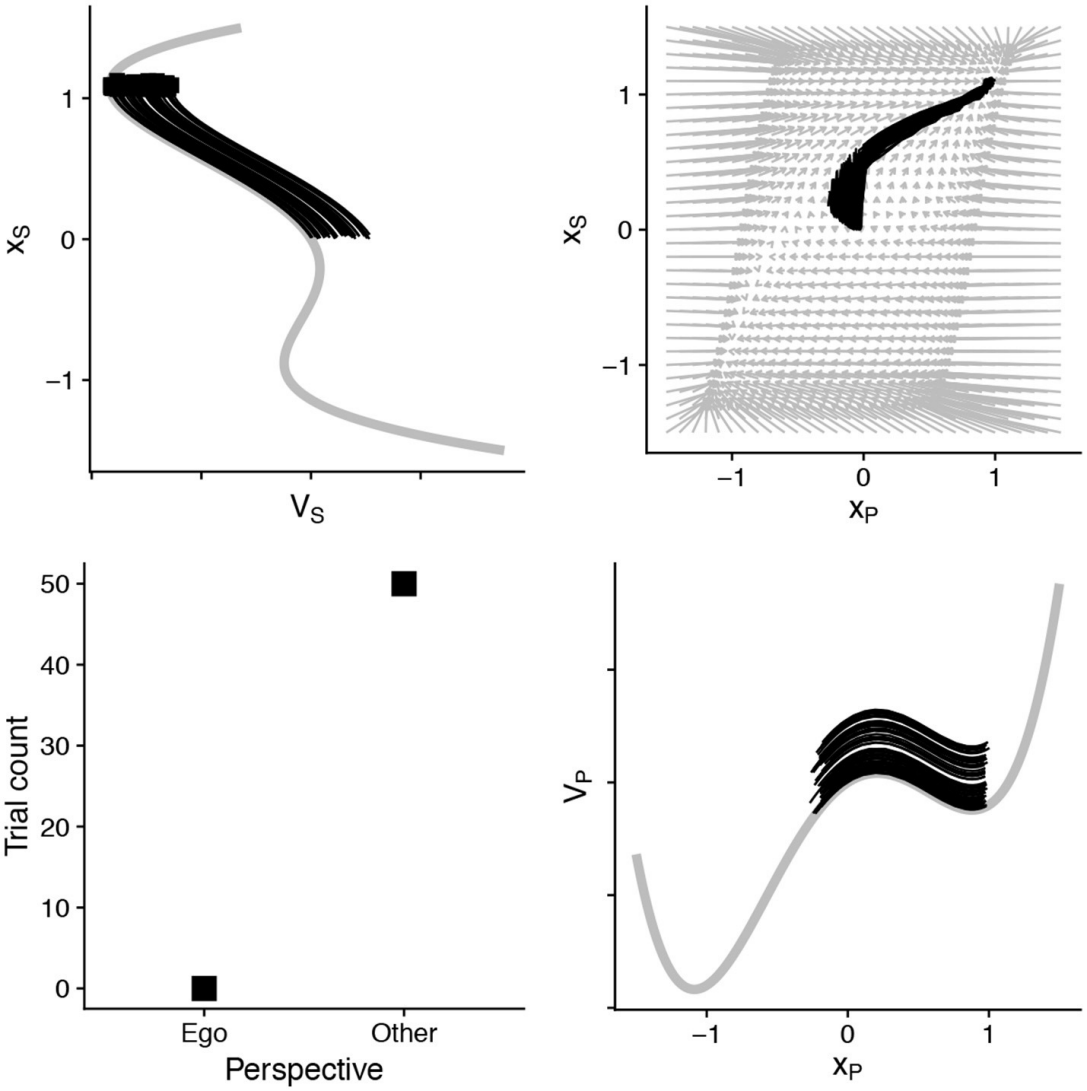
By assuming that slower process has an established “tilt” (“strategy”) and can recruit top-down control over lower-level processes (parameter α), other-centric responding can completely dominate—despite being half the speed and competing against a strong egocentric bias in the faster process. A relevant transformation of the vector field is also visible. Parameters in Table 2.

However, the high-level cognitive *strategy*, though slow to establish, can quickly dominate responses, even when egocentric responses are simpler (Duran et al., 2011). In the model, this can happen through two subtle changes. The “tilt” of the slower *x*_*S*_ variable can encode an established bias or strategy. This is shown in Figure 4. In addition, the parameter α can also modulate the dynamics of the fast and lower-level process (*x*_*P*_) (see the parameter set for Figure 4 in Table 2). A combination of parameters shows that perspective strategy, despite its slowness, can overcome and even “pull” fast processes into its relative regions.

**Figure 5:**
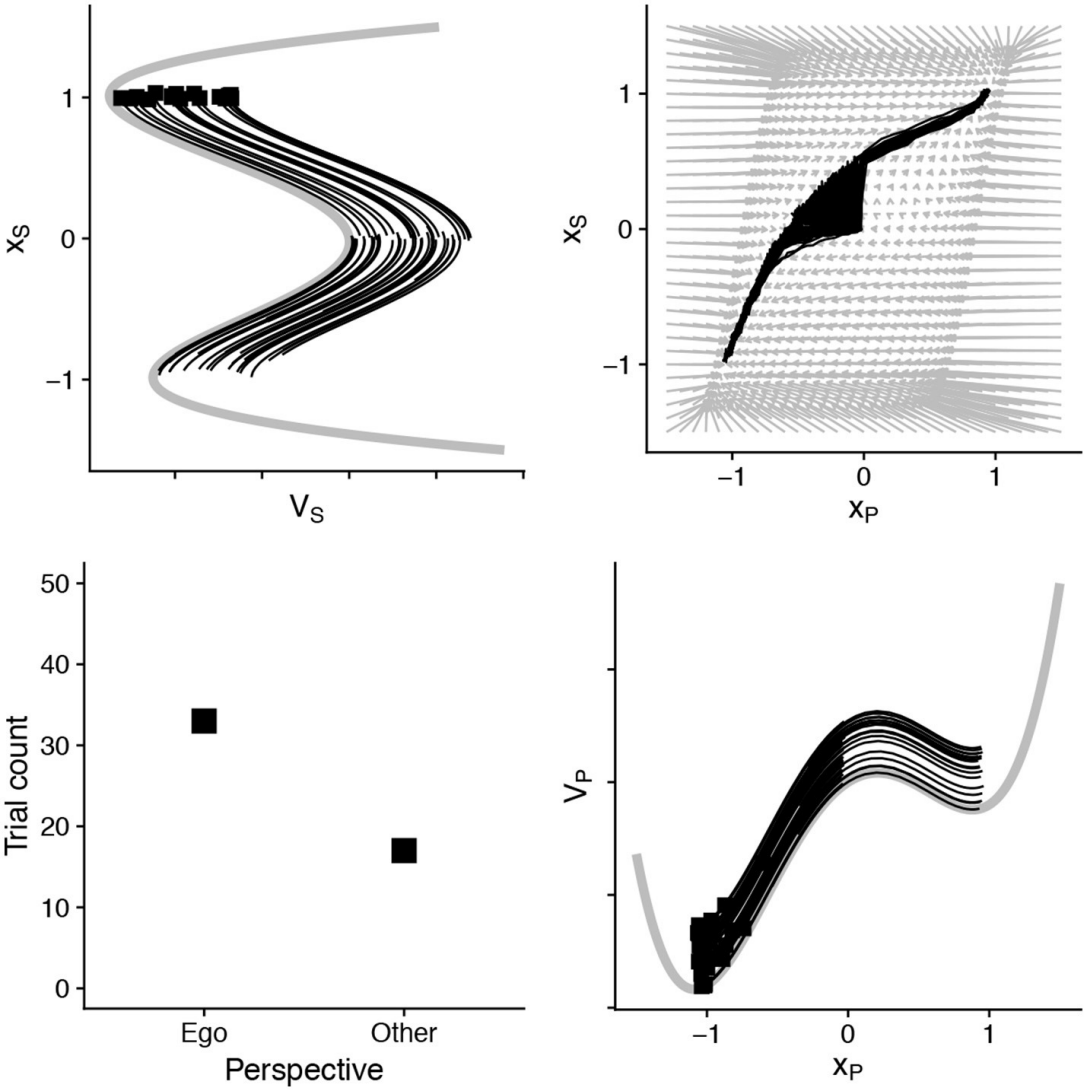
Under the condition that there is a bias for coherence, with α, β > 0, the vector field promotes common descent into consistent attractors—the processes facilitate each other. In this case, there is a slight tilt toward other-centric response (+1) for the slow process (*x*_*S*_) and again egocentric for the fast process (*x*_*P*_). There is mixed responding and the processes mutually reinforce the interpretations of each “decision”.

Similarly, if processes interact positively, the model predicts that decisions will be made more quickly and more confidently. Figure 5 illustrates this. If we assume that low and high-level processes pull each other—interpreted as a bias for coherence-the trajectories of the simulated decisions are fast and stable. The model therefore predicts that cue combinations may facilitate rapid perspective decisions, even in cases when other-centrism wins.

### Summary

This model obviously does not capture detailed cognitive processes. It is, however, an explicit formulation of informational dynamics at different speeds. Our gloss over the process in terms of perspective-taking allows us to think about slower and faster mechanisms as *interacting continually* rather than “coming first” or “coming second.” The model helps to visually and computationally frame the idea that during perspective-taking, overlapping processes are changing and influencing each other in order to stabilize a response. Task parameters, cognitive features, individual differences, etc., serve to modulate the vector field over which responses are made. And however simple, an explicit formulation of this kind motivates some further theoretical discussion, which we turn to next.

## Implications of the dynamic approach

Determining the relative contribution of the types of representations and processes involved in perspective-taking is tricky (Barr, 2014). The simulations of our dynamic model are meant to underscore a dissociation between the mapping of fast vs. slow processes onto the egocentric vs. other-centric perspectives. By modeling fast and slow processes as interacting continually, we have illustrated that other-centrism need not be second, and that indeed in some circumstances it can be fast—primarily because its effects can be *pre-established* in the cognitive system's potential (illustrated in the top-right panels in the prior figures).

This is consistent with findings that the design of a task may highlight some aspects over others and shape perspective-taking behavior accordingly. For example, an experimental paradigm for dialogue can emphasize egocentric, rapid processes (Keysar et al., 2000), but when it is fashioned to be more unscripted and interactive, it may reveal more rapid other-centric processes (Brown-Schmidt and Tanenhaus, 2008). Similarly, how mechanisms contribute is partly a function of the nature of a situation, or the demands of the task (e.g., in terms of time pressure or executive functioning). For instance, rushing participants can make it difficult to take another's perspective (Horton and Keysar, 1996), but if a task is framed as collaborative, they may deploy more robust tracking of common ground (Brown-Schmidt, 2009b). Similarly, in circumstances where the partner's perspective may be unambiguously computed and tracked, participants may be fast to adopt the partner's perspective (for discussion see Shintel and Keysar, 2009; Galati and Brennan, 2010). Indeed, cultural factors can also modulate the degree to which individuals are attentive to their partner's perspective (Wu and Keysar, 2007).

Altogether, perspective taking is robust human socio-cognitive capacity that is multiply constrained and supported. It is established by many processes, and how these processes are deployed will vary across contexts, depending on how information gets integrated over time. This view challenges a number of common assumptions that frame theoretical debates in cognitive science.

### The “default”

Our paper has focused on the debate regarding the status of an egocentric “default perspective.” As we have discussed, across all the domains reviewed, there is a common debate regarding this issue. Our view is that positing a default state is overly simplistic. It overlooks the fact that important aspects of our capacity to coordinate extend across not just milliseconds, but also minutes and longer moments. The fastest processes may occur only temporarily to buttress longer timescale mechanisms, such as the capacity for careful, deliberative social organization.

From the model, we can imagine a new sense of “default” here that focuses on *the most stable* aspect of our perspective-taking, rather than where we tarry momentarily *at first*. At the onset of a task, there is a slowly changing response as the cognitive system settles into a strategy. The default response may be best identified as the global minimum of the potential landscape (e.g., like that defined in Equation (1) above, and shown in Figure 1). This does not mean that responses are “slower” or “faster” in view of the default. Instead, their interaction and how they shape the probability of our responses is better identified as that default. Even here, it is important not to focus on speed on its own, but instead what that speeded response may be indicating about the perspective landscape that a task induces. We should not unduly theoretically prioritize these rapid processes, when both timescales play a critical role.

### Necessity and sufficiency

Some theories of the domains reviewed suggest that what we ought to determine are the unique necessary and sufficient conditions for generating target states such as joint actions (Bratman, 1993). In contrast, Tollefsen and Dale (2012) argue that developing overly rigid conceptions of these domains can limit the explanatory value of a theory (see Knoblich and Jordan, 2002; Vinson et al., 2017, for related discussion). For example, overly intellectualized cognitive conditions for joint actions restrict a theory's ability to connect to evolutionary, developmental, and computational considerations. Embracing a broad conception of perspective-taking permits researchers to identify the specific manifestation of this general conception in different species, different development stages, and different *tasks*. For example, Anderson's (2014) recent account of the neural basis for high-level cognition proposes that distinct *networks* of processes underlie various tasks. These networks can emerge in distinct ways, combining different sets of processes, from task to task. He analyzes brain region activity and gives each region a “functional fingerprint,” based on the distribution of that region's activity over different tasks. Tasks can thus be seen as networks of processes working together. Conversely, the same processes are mixed differently to sustain different tasks. For example, primary visual cortex is involved in many tasks, in a pattern that endows it with a “fingerprint” for that brain region; similarly, any task can be characterized as a distribution over the brain areas that contribute to its completion. Anderson shows this across a variety of domains. Such an account encourages researchers not to find necessary and sufficient conditions, but rather explore how perspective is supported by multiple flexible processes that are mixed to achieve the goals of a given task.

The model we presented illustrates this tension between necessity and sufficiency. The dynamics of perspective-taking emerge from a more fluid interaction among processes along the scales of Table 1. The cognitive components that support a given perspective interact dynamically and seek a stable state to render a response. In the case of spatial cognition, for example, information may combine rather quickly and simply and participants quickly take up their own perspective, all things being equal (Shelton and McNamara, 2001). Here the mechanisms are one's own spatial perception, proprioceptive information, and assumptions about the task. However if a subtle cue is perceived or activated in memory, such as a participant's lack of understanding (Schober, 2009), it may enhance or “tilt” the potential landscape, and even though the more strategic elements of a response are slower, they may be more entrenched and force the egocentric processes to follow. There is evidence that spatial cognition operates through such cooperating representations (Burgess, 2006). In neither of these cases is one or the other timescale “necessary” or “sufficient” alone.

### Beyond the cortex

The functional role of the environment—including both artifacts and conspecifics—is posited by distributed cognition and related domains (Hutchins, 1995; Clark, 1996; Clark and Chalmers, 1998). Human capacity for other-centrism need not be delimited by our cranium (Noë, 2009). For instance, our capacity to adopt perspectives distinct from our own sensorimotor perspective can be supported by external tools for visualization, ranging from hand-drawn diagrams to virtual reality technology, which readily permit representing and monitoring perspectives (of the self and of others; Lieberman, 2007). Particular social configurations can also amplify how external tools are used for purposes of perspective-taking. A provocative recent theory for language evolution is that only small neuroendocrinological changes may be needed to support strong social bonds in mammals (Syal and Finlay, 2011) and that such a change in our own species may have facilitated a strong social “glue.” Social cohesion of this sort would bring humans into strong and attentive groups, with our big primate brains then enhancing the socially structuring role of the environment and artifacts. These material objects have come to organize our mutual experience and mental processes (Tylén et al., 2016).

In our model, this intrinsic integration with the environment is possible by linking the constraints of a task or environment to the decision dynamics. Recent work on dynamic systems shows how this may be accomplished, with theoretically interesting results. Yoshimi (2012) identifies what he calls “active internalism” to describe open dynamical systems. In this view, the dynamics of the environment interact continually with dynamics that are intrinsic (or “internal”) to a cognitive system. This produces a new landscape that is shaped by this active interaction. Yoshimi (2012) supplies some elegant examples of this in the realms of consciousness and phenomenology. The process of perspective-taking could make use of this same modeling strategy—task parameters may be explicitly modeled as part of the landscape of responses.

### The traditional conception of “innate”

This approach also challenges conceptions of “innateness” and “specialization” that organize some of this literature (see discussion in Cohen and German, 2010). By our general definition, human perspective-taking is buttressed by a suite of processes that supports mutual responsiveness. Some of those processes may be specialized, at the functional or neurophysiological level (e.g., forward models and the mirror system, respectively, for supporting prediction about others' actions). But we would argue that human perspective-taking is not innate in the classic sense, of involving a unitary, genetically prescribed mechanism. Human perspective-taking is instead highly fluid. Faster and slower processes can work together or at different times, coming online under cognitive and task constraints. But the overarching outcome, the functionally relevant one for a social species, is task-relevant mutual responsiveness. Perspective-taking may be a highly robust social trait designed to be flexible and adaptive for the many coordinative tasks we face. Robustness, in the words of (Kitano, 2004, p. 827), is “the maintenance of specific functionalities of the system against perturbations, and it often requires the system to change its mode of operation in a flexible way. In other words, robustness allows changes in the structure and components of the system owing to perturbations, but specific functions are maintained.” Mutual responsiveness in humans may be so adaptive as a *general* social ability that it is maintained by the constellation of cognitive processes with which we are equipped.

The simple dynamic model portrays an interplay among processes, rather than a unitary innate perspective-taking mechanism. One possibility is that human perspective-taking reflects a layering of processes at various timescales, and that processes at a very fast timescale, such as human perceptuomotor responsiveness, may be a “cognitive homology” with other species, such as schooling fish. Humans have more layers of processing, expanding our capacities beyond the timescale of the here and now, and into more complex domains. Such a proposal, however, risks dangers of a teleological conception of human cognitive evolution (Penn et al., 2008). In any case, whether some or all of these (potentially layered) processes are innate is outside the scope of our present discussion.

## The emergentist approach to studying human other-centrism

We have argued that perspective-taking is emergent, from an interplay among diverse processes, in order for people to adapt to varied tasks. This thesis motivated a dynamical systems model. Though the model is simple, it illustrates the relationship among processes, and makes our claims computationally explicit. We argued in the preceding section that it promotes a different viewpoint on several issues of perspective-taking, such as the “default.”

In our model, perspective emerges from a stabilization of state variables, *x*_*P*_ and *x*_*S*_. This kind of stabilization is sometimes called a *collective variable*. In the realm of dynamical systems, a collective variable is a “coordinated pattern” that in turn “governs and constrains the behavior of the individual parts” (Kelso, 1995, pp. 8–9). It describes the macroscopic behavior of the system as a whole, and its functional properties at a coarser level of analysis. In other words, a perspective emerges from interactions among our cognitive processes, including basic (e.g., priming) and more specialized ones (e.g., forward models). Once established, a perspective can feed back onto those cognitive processes and constrain their performance. As a collective variable, perspective-taking constitutes the coordination dynamics of the interacting parts of a system that maintain responsiveness to other organisms through emergent and self-organized behavior. For example, in our model, as one aspect of the system stabilizes into one perspective or another, relationships among them can “pull” other parts of a system along. It is a capacity that is not defined by the *particular* configuration of the cognitive system, but instead by (temporarily) stable perspectives as its functional outcome.

Distilling observed social behaviors onto a collective variable—perspective and perspective-taking—may provide a tractable and generalizable way of modeling the dynamics of interaction. We believe this conception of perspective-taking, generally construed, encourages new or burgeoning theoretical and empirical questions. We address some of these below.

### Interactions among domains

If perspective-taking is a general process, emerging from many mechanisms and task constraints, then we should expect domains to interact. Seeing perspective-taking in such general terms leads to questions about how much interaction there is among types of perspective (beliefs, perceptions, emotions, and so on), and how broadly interactive they are. Such interactions may be facilitative. For example, social perspective-taking skills may *positively* interact with spatial tasks to counteract stereotype threat (Tarampi et al., 2016). Domain interaction may act in an inhibitory fashion. Sharing considerable history and co-presence among friends may lead to *disengagement* of certain perspective-taking processes (Savitsky et al., 2011), and indeed false or failed perspective may lead social judgments astray (Eyal et al., 2018)^5^. Some cognitive neuroscience research suggests that these domains may have both shared and distinct substrates, suggesting that interaction is possible, but that mechanisms may also have distinct bases (e.g., for empathy vs. ToM: Kanske et al., 2015). In general, there is much work left to do in studying the way in which mechanisms support significant task transfer effects. For example, a massive literature in social cognition indicates there are spillover effects in social positivity when certain perspective features align (e.g., morals: Feinberg and Willer, 2015; e.g., behaviors: Lakin et al., 2003).

### Integrative modeling

If there are both shared and distinct subsystems, then developing a broader understanding of the integration among elements of perspective is key. One way forward is integrative computational models. The model we presented here may be a start to dynamical systems modeling (based on Tuller et al., 1994). The model is simple, but makes more explicit our hypotheses about timescale interactions. Recent work has sought to put aspects of perception and cognition into such attractor systems (e.g., Frank et al., 2009). By further exploring parameters that may extend our two-dimensional system, it may be possible to render predictions about more specific task contexts. Neural network models may supply another arena to build in multiple constraints and fit data trends in different interactive domains, such as spatial perspective-taking (Duran et al., 2016). Such computational models allow explicit formulation of collective variables, and exploration of their dynamic properties.

We have noted that the account we develop here implies that perspective-taking is not a unitary process—not a single self-contained architecture, but rather a varied cognitive solution emerging from a mixture of processes under tasks. A possible critique of this account is that its value will depend upon exploring how theoretically productive it is. Our discussion in this section, and in the prior section, elaborates on some directions that may be considered productive. However, the integrative modeling approach may also facilitate this productivity. Even if one disagrees with our non-unitary stance, the current state of affairs is one of moderate fractionation (“non-unitariness”). We highlighted this in the introduction to our paper. This fractionation motivated our review that perspective-taking is central to many fields of cognitive science, but these are rarely systematically integrated. So even leaving these domains alone, so to speak, we are left with a non-unitary *situation*. Our discussion highlights their connections through *shared mechanisms*. Integrative modeling may help form a computational basis for bridging these areas, and understanding which mechanisms support perspective-taking tasks. In an ironic sense, embracing non-unitary explanations can help develop a broader understanding of specific domains.

### Dynamic structure

It is still generally unknown how perspective-taking unfolds. Some significant progress in psycholinguistics has been achieved using eye-tracking measurements (e.g., fixations on target referents), which permits researchers to identify the time course with which perspectives are considered (e.g., Wu et al., 2013). In addition, brain imaging techniques and other cognitive tasks have revealed that perspectives may indeed overlap and contribute jointly during some tasks, such as spatial cognition and navigation (Burgess, 2006; Gagnon et al., 2014). It may be that a distinct signature in EEG spectral properties may mark the emergence of social coordination and related phenomena (Tognoli and Kelso, 2015). Another related behavioral method is mouse-tracking, which yields measures of arm movements while perspective decisions are made (Duran et al., 2011; cf. Spivey and Dale, 2006; Spivey, 2007; Freeman and Ambady, 2011).

Such measures can inform many still open questions about the dynamic structure of perspective-taking. How non-linear is perspective-taking change? Does it occur in a rapid shift, or do perspectives compete more slowly? How does perspective selection stabilize under different task constraints? Exploring dynamic instability is compatible with our view of perspective-taking as a collective variable; collective variables are, by definition, the dimension on which self-organized, spontaneous change in patterns occurs in the system (e.g., qualitative shifts, such as fluctuations from egocentric to other-centric orientation, or vice-versa).

One dynamic behavioral index that can capture the multi-scale structure of perspective-taking is complexity matching. This framework for capturing coordination comes from the domain of statistical physics, in which complex network coupling is modeled for maximal information transfer (West et al., 2008). Evidence of complexity matching in interpersonal coordination has been recently found in terms of both perceptuomotor behavior and speech, with the degree of complexity matching varying under different task constraints (Marmelat and Delignières, 2012; Abney et al., 2014).

Seeking such dynamic behavioral indices, including in brain imaging and implicit behavioral measures, could better capture the emergence of a perspective strategy and reveal how timescales behave. For example, complexity matching of speech during communication reveals which timescales are more or less affected by a task (Ramirez-Aristizabal et al., 2018). These distinct characteristics of temporal structure in speech are hypothesized to reflect how two people are mutually organizing their behavior and cognition.

### Cue integration

One broad set of answers that a dynamic approach might supply is to the question of how and when different perspective cues are integrated. For example, subtle and simple instructions to participants can alter the probability distribution for perspectives in a spatial task, and the dynamics of perspective responses (Duran and Dale, 2014). In related tasks, the alignment of a spatial configuration with social perspectives can also radically alter perspective strategy (Galati and Avraamides, 2015; Galati et al., 2017). This suggests that interaction among cues might alter cognitive dynamics in surprisingly profound ways. Taking a dynamic approach may expand such questions, and develop a deeper understanding of how humans integrate particular pieces of information about a task partner.

## Conclusion

We have argued that perspective and perspective-taking should be understood as an integration of various processes, and across interacting timescales. The result is a “big tent” approach to perspective that discourages unitary explanations anchored to one domain, or separately in several domains. A dynamical systems approach, illustrated by a simple model here, supports a conception of perspective and perspective-taking as stable behavioral strategies generated by *many* (potentially interacting) factors, not just domain-specific mechanisms. It leads to new methods and new questions. Though space restricts us here, we would argue that it has other benefits than just those described above. A general conception is broad enough to link to comparative and evolutionary questions. It is more flexible for exploring graded developmental trajectories as perspective-taking emerges in children. It may motivate multiple distinct computational paths for perspective-taking in epigenetic robotics and other frameworks of artificial intelligence. It may also help link subdomains, and facilitate new theory development.

## Author contributions

AG and RD led multiple discussions, literature review, and writing. The manuscript emerged from a laboratory reading group that included all co-authors. All co-authors contributed to the writing and revision of the article.

### Conflict of interest statement

The authors declare that the research was conducted in the absence of any commercial or financial relationships that could be construed as a potential conflict of interest.

## Footnotes

^1^https://somby.ceu.edu/jam.

^2^It should be remarked that ToM has a somewhat controversial status, with many researchers challenging the existence or explanatory need of such a high-level kind of process (for some discussion, see Smith, 1999). It is nevertheless a highly influential notion, and so we integrate it here.

^3^There has been some integration. Conversation can be seen as joint action (Clark, 1996), and theory of mind and conversational processes can be investigated as tightly intertwined (Rosnay and Hughes, 2006). The common thread of perspective-taking, however, is not as frequently studied in more general terms.

^4^All code to regenerate these simulations can be found at the URL github.com/racdale/simple-perspective-model.

^5^Indeed a critical aspect of perspective-taking and interaction is the capacity to *decouple* and coordinate more complexly with another person. We cannot elaborate on this issue here, but some intriguing recent work on measuring and describing this tendency is worth considering (Fusaroli et al., 2012; Raczaszek-Leonardi et al., 2014). In this work, the dyad (or group) is seen as coordinating more than just perspective, but also aspects of overt behavioral and environmental constraints to adapt to and carry out structured tasks.
